# High-flow nasal cannula versus conventional oxygen therapy in acute COPD exacerbation with mild hypercapnia: a multicenter randomized controlled trial

**DOI:** 10.1186/s13054-022-03973-7

**Published:** 2022-04-15

**Authors:** Jingen Xia, Sichao Gu, Wei Lei, Jihua Zhang, Hui Wei, Chao Liu, Han Zhang, Rongli Lu, Liqiong Zhang, Mingyan Jiang, Chao Hu, Zhenshun Cheng, Chaojie Wei, Yusheng Chen, Fengfeng Lu, Min Chen, Hong Bi, Hui Liu, Cunzi Yan, Hong Teng, Yang Yang, Chen Liang, Yanlei Ge, Pengguo Hou, Jialin Liu, Weiwei Gao, Yi Zhang, Yingying Feng, Cheng Tao, Xu Huang, Pinhua Pan, Hong Luo, Chunmei Yun, Qingyuan Zhan

**Affiliations:** 1 Department of Pulmonary and Critical Care Medicine, Center of Respiratory Medicine, China-Japan Friendship Hospital, No. 2 East Yinghua Road, Chaoyang District, Beijing, 100029 China; 2 National Center for Respiratory Medicine, Beijing, China; 3 Institute of Respiratory Medicine, Chinese Academy of Medical Sciences, Beijing, China; 4 National Clinical Research Center for Respiratory Diseases, Beijing, China; 5 School of Biological Science and Medical Engineering, Beihang University, Beijing, China; 6 Department of Pulmonary and Critical Care Medicine, The First Affiliated Hospital of Soochow University, Suzhou, China; 7 Department of Pulmonary and Critical Care Medicine, The People’s Hospital of Yuxi City, The Sixth Affiliated Hospital of Kunming Medical University, Yuxi, China; 8 Department of Pulmonary and Critical Care Medicine, Inner Mongolia People’s Hospital, No. 20 Zhaowuda Road, Hohhot, 010017 China; 9 Department of Pulmonary and Critical Care Medicine, The Second Xiangya Hospital, Central South University, No.139 Renmin Middle Road, Changsha, 410011 China; 10 Department of Pulmonary and Critical Care Medicine, Xiangya Hospital, Central South University, No.87 Xiangya Road, Changsha, 410008 China; 11 Department of Pulmonary and Critical Care Medicine, Xiangtan Central Hospital, Xiangtan, China; 12 Department of Pulmonary and Critical Care Medicine, Zhongnan Hospital of Wuhan University, Wuhan, China; 13 Wuhan Research Center for Infectious Diseases and Cancer, Chinese Academy of Medical Sciences, Wuhan, China; 14 Department of Pulmonary and Critical Care Medicine, Fujian Provincial Hospital, Fuzhou, China; 15 Department of Pulmonary and Critical Care Medicine, Calmette Hospital and The First Hospital of Kunming, Kunming, China; 16 Department of Pulmonary and Critical Care Medicine, The First Affiliated Hospital of Xinjiang Medical University, Urumqi, China; 17 Department of Pulmonary and Critical Care Medicine, Sichuan Provincial People’s Hospital, University of Electronic Science and Technology of China, Chengdu, China; 18 Chinese Academy of Sciences Sichuan Translational Medicine Research Hospital, Chengdu, China; 19 Chinese Alliance for Respiratory Diseases in Primary Care, Beijing, China; 20 Department of Pulmonary and Critical Care Medicine, North China University of Science and Technology Affiliated Hospital, Tangshan, China; 21 Department of Pulmonary and Critical Care Medicine, The Third People’s Hospital of Datong, Datong, China; 22 Department of Critical Care Medicine, Ruijin Hospital, Shanghai JiaoTong University School of Medicine, Shanghai, China; 23 Department of Respiratory Medicine, The Second Affiliated Hospital of Zhengzhou University, Zhengzhou, China

**Keywords:** High-flow nasal cannula, Respiratory support, Respiratory insufficiency, Pulmonary disease, Chronic obstructive, Hypercapnia

## Abstract

**Background:**

High-flow nasal cannula (HFNC) can improve ventilatory function in patients with acute COPD exacerbation. However, its effect on clinical outcomes remains uncertain.

**Methods:**

This randomized controlled trial was conducted from July 2017 to December 2020 in 16 tertiary hospitals in China. Patients with acute COPD exacerbation with mild hypercapnia (pH ≥ 7.35 and arterial partial pressure of carbon dioxide > 45 mmHg) were randomly assigned to either HFNC or conventional oxygen therapy. The primary outcome was the proportion of patients who met the criteria for intubation during hospitalization. Secondary outcomes included treatment failure (intolerance and need for non-invasive or invasive ventilation), length of hospital stay, hospital cost, mortality, and readmission at day 90.

**Results:**

Among 337 randomized patients (median age, 70.0 years; 280 men [83.1%]; median pH 7.399; arterial partial pressure of carbon dioxide 51 mmHg), 330 completed the trial. 4/158 patients on HFNC and 1/172 patient on conventional oxygen therapy met the criteria for intubation (*P* = 0.198). Patients progressed to NPPV in both groups were comparable (15 [9.5%] in the HFNC group vs. 22 [12.8%] in the conventional oxygen therapy group; *P* = 0.343). Compared with conventional oxygen therapy, HFNC yielded a significantly longer median length of hospital stay (9.0 [interquartile range, 7.0–13.0] vs. 8.0 [interquartile range, 7.0–11.0] days) and a higher median hospital cost (approximately $2298 [interquartile range, $1613–$3782] vs. $2005 [interquartile range, $1439–$2968]). There were no significant differences in other secondary outcomes between groups.

**Conclusions:**

In this multi-center randomized controlled study, HFNC compared to conventional oxygen therapy did not reduce need for intubation among acute COPD exacerbation patients with mild hypercapnia. The future studies should focus on patients with acute COPD exacerbation with respiratory acidosis (pH < 7.35). However, because the primary outcome rate was well below expected, the study was underpowered to show a meaningful difference between the two treatment groups.

*Trial registration*: NCT03003559. Registered on December 28, 2016.

**Supplementary Information:**

The online version contains supplementary material available at 10.1186/s13054-022-03973-7.

## Background

Non-invasive positive pressure ventilation (NPPV) can significantly reduce the need for intubation and the in-hospital mortality rate among patients with acute chronic obstructive pulmonary disease (COPD) exacerbation with respiratory acidosis [1–3]. However, it is currently not recommended for use in mild hypercapnic acute COPD exacerbations without acute respiratory acidosis (pH ≤ 7.35) [4]. Therefore, conventional oxygen therapy (COT) is the most commonly used standard treatment for these patients [2]. However, COT is often associated with a variable fraction of inspired oxygen (FiO_2_), dryness of the nose and mouth, nasal mucosal bleeding, and intolerance. Moreover, several previous studies have reported that 7–15% of COPD patients who experience acute exacerbation with COT require upgrading to invasive mechanical ventilation [5–7], which is an indicator of an important increased mortality risk [1–4].

High-flow nasal cannula (HFNC) is a novel modality of respiratory support technology that has emerged for use in adult patients with acute respiratory failure over the past decade [8]. It has been proved to have several remarkable physiological advantages [8–12]. Several recent meta-analyses [11–13] found that HFNC can reduce the risk of endotracheal intubation in patients with acute hypoxic respiratory failure when compared with COT. It is noteworthy that most randomized controlled trials exploring the use of HFNC in acute respiratory failure have excluded hypercapnic patients [14–16].

Recently, physiological studies of small samples of acute exacerbations and stable COPD patients found that short-term (within 2 h) application of HFNC could effectively decrease the arterial partial pressure of carbon dioxide (PaCO_2_) (by 4%-12%) [17–20], reduce the physiological dead space [17–21], attenuate the work of breathing [22–24], and improve airway clearance [25]. Two randomized controlled trials [26, 27] reported that compared with long-term oxygen therapy, long-term (6 weeks to 1 year) HFNC therapy can further improve the quality of life and reduce the risk of readmission due to acute exacerbations among patients with stable COPD. However, the effect of HFNC on clinical outcomes in patients with acute COPD exacerbation with mild hypercapnia remains uncertain.

Therefore, in this randomized controlled trial, we hypothesized that compared to COT, HFNC would reduce the need for intubation for acute COPD exacerbation patients with mild hypercapnia (pH ≥ 7.35, PaCO_2_ > 45 mmHg).

## Methods

### Study design

This randomized clinical trial was conducted in the general respiratory wards of 16 tertiary hospitals in China from July 2017 to December 2020. The ethics committee of each hospital approved the study protocol. An investigator at each hospital was responsible for daily patient screening, selection, randomization, and electronic data recording, as per the study protocol. The investigators did not participate in the daily medical care of the enrolled patients. All patients or their relatives provided written informed consent.

### Study participants

All patients admitted to the hospital with a main diagnosis of acute COPD exacerbation according to GOLD criteria were enrolled if they had mild hypercapnia (pH ≥ 7.35 and PaCO_2_ > 45 mmHg) at admission.

The main exclusion criteria were age > 85 years, Glasgow Coma Scale score < 12, home NPPV, obstructive sleep apnoea syndrome, excessive airway secretions that are difficult to drain, hemodynamic instability (systolic blood pressure < 90 mmHg, mean blood pressure < 65 mmHg, or blood pressure lower than the baseline value of 40 mmHg, and a lactate level > 2 mmol/L among patients receiving vasoactive drugs), severe arrhythmia or acute coronary syndrome, respiratory and cardiac arrest, palliative care, refusal to be included in this study, or participation in other studies.

At inclusion, demographic variables (age, sex, acute physiology and chronic health evaluation [APACHE] II score, and comorbidities) were recorded. In the 72 h after randomization, the following variables were recorded: arterial blood gas, oxygen saturation (SpO_2_), Borg dyspnoea scale score, subjective airway (oral, nasal, and throat) dryness score (0–10 scale, on which 0 is normal and 10 is severest form of dryness), laboratory biochemical indexes, and vital signs. Patients were followed up for 90 days after randomization.

### Randomization

The randomization scheme was computer generated using a centralized Web-based management system in permuted blocks of four or six participants, with stratification according to the center. The patient ID number was required for randomization, and each ID number was randomized only once. The patients were randomly divided into a HFNC group (intervention group) or a COT group (control group) in a 1:1 ratio.

### Interventions

All patients in the HFNC group received HFNC therapy using Airvo-2™ equipment (Fisher & Paykel Healthcare, Auckland, New Zealand). According to the published literature [20–22], the initial HFNC flow rate was set to 25 L/min to improve ventilatory function and gradually increased (5–10 L/min each time) to patient’s maximum tolerance. The FiO_2_ was adjusted to maintain a SpO_2_ between 90 and 95%. The inhaled gas temperature (31–37 °C) was set at the patient’s maximum tolerance level. HFNC therapy was recommended for use as long as possible every day. HFNC treatment was withdrawn once the flow rate and FiO_2_ were lower than 20 L/min and 0.3, respectively.

Patients in the COT group received continuously low flow oxygen therapy via a nasal cannula. Moreover, the oxygen flow rate in the nasal cannula (1–5 L/min) was titrated to maintain an SpO_2_ of 90–95%.

### Outcomes

The primary outcome was the proportion of patients who met the criteria for intubation (need for intubation) during hospitalization. The need for intubation was a more objective and uniform variable, avoiding inconsistencies associated with intubation affected by multiple factors, including ICU bed and equipment availability, physician’s experience, etc. The criteria for intubation and invasive mechanical ventilation included intolerance of NPPV, severe acute respiratory failure in which NPPV was difficult to correct, pH < 7.25 accompanied by a progressive increase in PaCO_2_, respiratory or cardiac arrest, loss of consciousness and delirium, massive aspiration, inability to clear airway secretions, severe hemodynamic instability, severe arrhythmia, and life-threatening hypoxemia.

The secondary outcomes were the rate of treatment failure (intolerance, need for intubation, or NPPV), daily duration of HFNC and COT treatment during the first 7 days (the day of randomization was called day one), proportion of patients upgraded to NPPV, actual intubation rate, length of hospital stay, hospital cost (the sum of all expenses incurred during hospitalization, including bed cost, instrumental examination cost, lab investigation cost, treatment cost, drug cost, nursing care cost, and medical consumables, etc., but excluding the cost of HFNC circuits), mortality rate in the hospital and at day 90, and the readmission rate after discharge due to acute exacerbations at day 90. The criteria for NPPV treatment included worsening respiratory acidosis (pH < 7.35), severe dyspnoea, respiratory muscle fatigue, or increased work of breathing (e.g., accessory respiratory muscle score ≥ 3) [28], and severe hypoxemia (PaO_2_ < 50 mmHg). The respiratory support modality (nasal cannula, NPPV, or invasive mechanical ventilation) after treatment failure in both groups was determined by consultation with the attending physician and the patient.

Exploratory outcomes included blood gas, Borg dyspnoea scale score and airway dryness score at 2, 24, 48, and 72 h after randomization, and adverse events.

### Statistical analysis

According to a previous publication [6], the proportion of patients meeting the criteria for intubation was 11.3% among patients with mild acute COPD exacerbation (pH > 7.35) using nasal cannula. Due to the absence of previous clinical trials that investigated the use of HFNC for milder acute COPD exacerbations, assuming the proportion of patients meeting the criteria for intubation of 3% after application of HFNC, a total sample size of 328 patients was required to achieve a power of 80% to detect the difference at a two-sided alpha level of 0.05, after accounting for a loss to follow-up rate of 10%.

All analyses were conducted according to the intention-to-treat principle. Data for continuous variables with normal and skewed distributions were reported as means or medians and standard deviations or interquartile ranges (IQRs), respectively. Categorical variables were reported as frequencies and percentages. For group comparisons of efficacy and safety endpoints, the student *t*-test was used for variables with normal distributions, the Mann–Whitney *U* test was applied to skewed variables, and Pearson’s Chi-squared test or Fisher’s exact test was performed for categorical variables. We used time-to-event methods, including Kaplan–Meier curves and log-rank tests, to compare the overall survival within 90 days after intervention and the time to readmission within 90 days after randomization between the intervention and control groups. We applied a post hoc random-effect regression model to adjust for center for the primary outcome. The cumulative incidence function and Gray’s test were used to consider deaths as competing events to evaluate the difference of time to readmission for acute exacerbation between two groups.

All analyses were performed using SAS version 9.4 (SAS Institute Inc., Cary, NC, USA). Statistical items with a two-sided *P*-value < 0.05 were considered statistically significant.

## Results

### Study participants

**Fig. 1:**
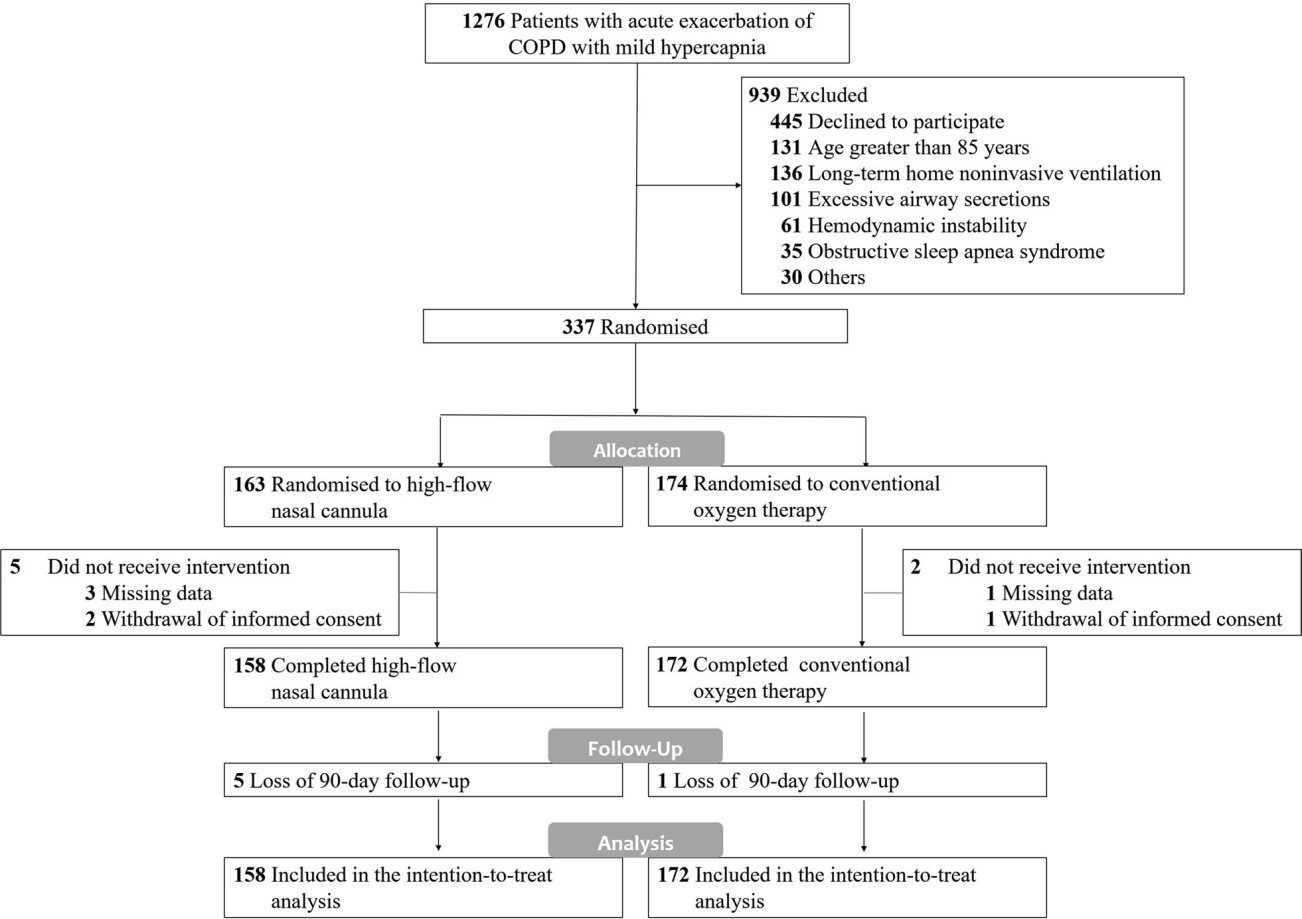
Flow of participants through study

During the study period, 1276 patients with acute COPD exacerbation with mild hypercapnia were identified, 337 (median age, 70.0 years; 280 men [83.1%]) of whom were randomized. Seven patients were secondarily excluded because they had missing data for the primary outcome (*n* = 4) or withdrew informed consent (*n* = 3). The remaining 330 patients were included in the analysis: 158 in the HFNC group and 172 in the COT group (Fig. 1 and Table 1).

**Table 1:** Baseline patient characteristics

Characteristic	No. (%)	
	High-flow nasal cannula group (*n* = 158)	Conventional oxygen therapy group (*n* = 172)
*Characteristics of the patients at admission*		
Age, median (IQR), y	70.0(65.0–75.0)	69.0(63.5–74.5)
Men, No. (%)	140(88.6%)	137(79.7%)
Body mass index*, median (IQR), kg/m^2^	21.0(18.7–23.3)	21.0(18.7–23.3)
APACHE II score, median (IQR)	10.0(7.0–13.0)	10.0(7.0–13.5)
Symptoms at admission, No. (%)		
Dyspnoea	149(94.3%)	170(98.8%)
Cough	143(90.5%)	157(91.3%)
Wheeze	118(74.4)	138(80.2%)
Sputum production	101(63.9%)	91(52.9%)
Fever	24(15.2%)	24(14%)
Comorbidities, No. (%)		
Hypertension	54(34.2%)	65(37.8%)
Diabetes mellitus	16(10.1%)	13(7.6%)
Asthma	11(7.0%)	8(4.7%)
Chronic heart failure	7(4.4%)	12(7.0%)
Bronchiectasis	7(4.4%)	10(5.8%)
Current smoker, No. (%)	34(21.5%)	35(20.3%)
FEV1% predicted, No	59	67
Median (IQR)	32.5(24.8–42.1)	32.0(24.6–43.1)
FEV1/FVC% predicted, No	60	67
Median (IQR)	41.6(33.0–51.3)	42.6(34.6–51.6)
Prior use of long-term oxygen therapy, No. (%)	37(23.4%)	40(23.3%)
*Physiological characteristics at randomization*		
Body temperature, median (IQR), °C	36.5(36.4–36.8)	36.6(36.4–36.8)
Respiratory rate, median (IQR), breaths/min	21.0(20.0–23.0)	21.0(20.0–23.0)
Heart rate, median (IQR), beats/min	85.0(78.0–98.0)	88.5(78.5–100.0)
Mean blood pressure, median (IQR), mmHg	95.0(87.3–102.7)	96.0(88.7–102.8)
SpO_2_, median (IQR), %	93.0(89.0–96.0)	92.0(88.0–96.0)
Nasal cannula, No. (%)	89(56.3%)	98(57.0%)
O_2_ flow, median (IQR), L/min	2.0(2.0–3.0)	2.0(2.0–3.0)
Borg scale score, median (IQR), units	4.0(3.0–5.0)	4.0(3.0–5.0)
pH, median (IQR), units	7.40(7.37–7.42)	7.40(7.37–7.43)
PaCO_2_, median (IQR), mmHg	50.4(47.3–56.3)	51.7(47.6–58.0)
PaO_2_, median (IQR), mmHg	70.4(57.0–83.0)	68.0(56.0–83.7)
Bicarbonate, median (IQR), mmol/L	31.3(28.3–34.9)	31.8(29.0–35.2)
White blood cell, median (IQR), × 10^9^/L	6.9(5.5–9.4)	6.9(5.8–8.8)
C-reactive protein, median (IQR), mg/L	10.0(3.9–29.1)	8.7(4.1–30.9)

*APACHE II* acute physiology and chronic health evaluation II, *FEV1* forced expiratory volume in one second, *FVC* forced vital capacity, *IQR* interquartile range, *PaCO*_*2*_ arterial partial pressure of carbon dioxide, *PaO*_*2*_ arterial partial pressure of oxygen, *SpO*_*2*_ oxygen saturation

*Calculated as weight in kilograms divided by height in meters squared

### HFNC treatment

The initial median settings in the HFNC group were as follows: flow rate, 30.0 L/min (IQR, 25.0–40.0); FiO_2_, 0.32 (IQR, 0.3–0.4); and gas temperature, 31.0 °C (IQR, 31.0–34.0) (Additional file 1: Table S1). Within 7 days of randomization, the total median duration of HFNC treatment was 82.0 h (IQR, 44.0–137.0), which was shorter than that of nasal cannula in the COT group (111.0 h [IQR, 66.0–148.5]) (*P* = 0.005) (Additional file 1: Table S1). Moreover, the daily treatment duration of HFNC was also shorter than that of nasal cannula in the COT group within 7 days after randomization (Additional file 2: Table S2).

### Primary outcome

Compared to the COT group, the HFNC group had a similar proportion of patients who met the criteria for intubation (2.5% [*n* = 4] in the HFNC group vs. 0.6% [*n* = 1] in the COT group, *P* = 0.198 without center random effect, and *P* = 0.186 after adjustment for center random effect) (Table 2).

**Table 2:** Primary and secondary outcomes

Characteristic	No. (%)		Absolute difference, % (95%CI)	*P*
	High-flow nasal cannula group (*n* = 158)	Conventional oxygen therapy group (*n* = 172)		
*Primary outcome*				
Criteria for intubation, No. (%)	4 (2.5%)	1 (0.6%)	1.95 (− 0.8–4.7)	0.198*
*Secondary outcome*				
Treatment failure, No. (%)	25 (15.8%)	25 (14.5%)	1.29 (− 6.5–9.0)	0.745*
Intubation, No. (%)	3 (1.9%)	1 (0.6%)	1.95 (− 0.8–4.7)	0.353*
NPPV, No. (%)	15 (9.5%)	22 (12.8%)	− 3.3 (− 10.1–3.5)	0.343*
Duration of NPPV, median (IQR), days	6.0 (2.0–10.0)	5.5 (4.0–8.0)	1.0 (− 2.7–4.7)	0.780^†^
Mortality in hospital, No. (%)	0 (0%)	1 (0.6%)		> 0.999*
Mortality at day 90, No. (%)	5/153 (3.3%)	5/171 (2.9%)	0.34 (− 3.4–4.1)	> 0.999*
Length of hospital stay, median (IQR), days	9.0 (7.0–13.0)	8.0 (7.0–11.0)	1.0 (0.0–2.0)	0.021^†^
Hospital cost, median (IQR), $	2298 (1613–3782)	2005 (1439–2968)	265 (− 104–632)	0.006^†^
Readmission rate at day 90, No. (%)	25/153 (16.3%)	23/170 (13.5%)	2.8 (− 5.0–10.6)	0.478*

*NPPV* noninvasive positive pressure ventilation, *IQR* interquartile range

*Fisher exact test or χ^2^

^†^Mann–Whitney *U*

### Secondary outcomes

There was no significant difference in the rate of treatment failure between the groups (15.8% [*n* = 25] vs. 14.5% [*n* = 25] in the HFNC and COT groups, respectively; *P* = 0.745) (Table 2). The most common reason for treatment failure in the HFNC group was intolerance to HFNC treatment (*n* = 13, 52.0%), and the most common reason for treatment failure in the COT group was need for NPPV (*n* = 18, 72%) (Table 2).

Patients upgraded to NPPV in both groups were comparable (15 [9.5%] in the HFNC group vs. 22 [12.8%] in the COT group; *P* = 0.343) (Table 2). However, compared to the COT group, the median duration from randomization to the start of NPPV treatment was longer in the HFNC group (4.0 [IQR, 3.0–8.0] vs. 2.0 [IQR, 1.0–5.0] days; *P* = 0.060). The median total duration of NPPV treatment was similar between the groups (HFNC 6.0 days vs. COT 5.5 days; *P* = 0.780) (Table 2).

In this study, a total of five patients reached the predetermined criteria for intubation, of whom four were directly intubated and treated with invasive ventilation (HFNC group, *n* = 3; COT group, *n* = 1), and one patient in the HFNC group was successfully treated with NPPV. There was no significant difference in the actual intubation rate between the two groups (*P* = 0.353) (Table 2).

There were no deaths in the HFNC group during hospitalization, and one patient in the COT group died of ventilator-associated pneumonia and septic shock after intubation (Table 2). Compared with the COT group, patients in the HFNC group had a significantly longer median length of hospital stay (9.0 [IQR, 7.0–13.0] vs. 8.0 [IQR, 7.0–11.0] days, *P* = 0.021). HFNC increased the median hospital cost by about 14.6% compared to the COT group (approximately $2298 [IQR, $1613–$3782] vs. $2005 [IQR, $1439–$2968]; *P* = 0.006) (Table 2).

**Fig. 2:**
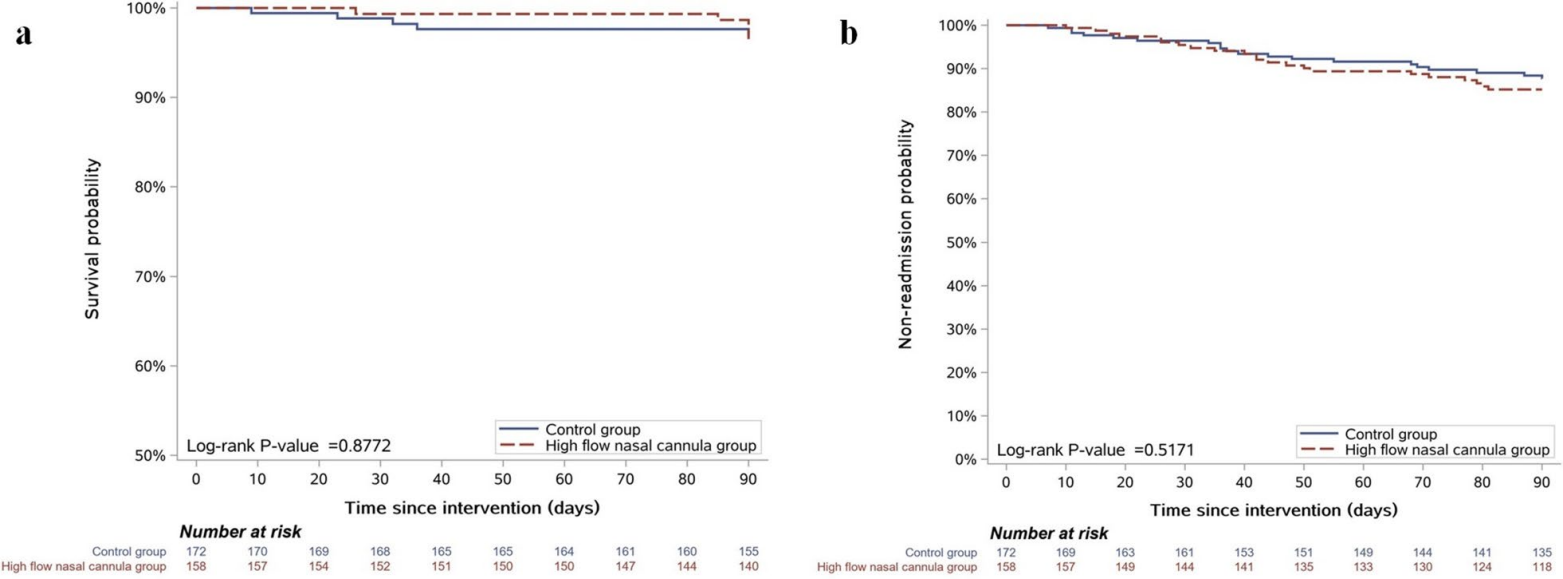
Kaplan–Meier analysis of time since intervention to death (**a**) and time since intervention to readmission (**b**) during 90-day follow-up period

**Fig. 3:**
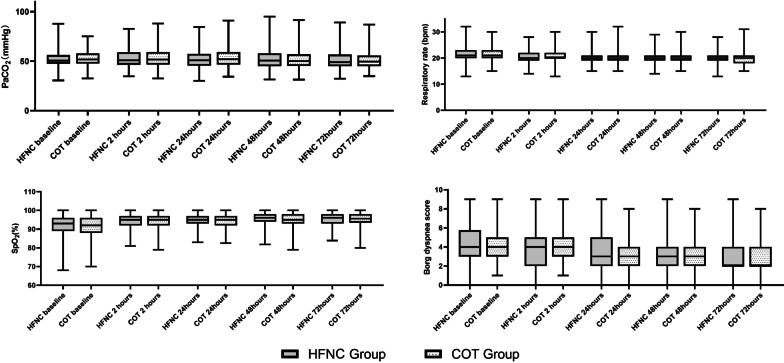
Changes of PaCO_2_, respiratory rate, SpO_2_ and Borg dyspnea score within 72 h after randomization between the two groups. Data are presented as median (interquartile range). *COT* conventional oxygen therapy group, *HFNC* high-flow nasal cannula group, *PaCO*_*2*_ arterial partial pressure of carbon dioxide, *SpO*_*2*_ oxygen saturation

During the 90-day follow-up period after randomization, the mortality rate was not significantly different between the two groups (3.3% vs. 2.9% in the HFNC and COT groups, respectively; *P* > 0.999) (Table 2 and Fig. 3). The proportions of readmission due to exacerbation in both groups were 16.3% and 13.5% in the HFNC and COT groups, respectively, with no statistical difference (*P* = 0.478) (Table 2 and Fig. 2). Considering deaths as competing events, time to readmission for acute exacerbation was also similar in two groups (Gray’s test *P* = 0.3979, Additional file 4: Figure S1).

### Exploratory outcomes

Within 72 h of randomization, there were no significant differences in PaCO_2_, PaO_2_, SpO_2_, respiratory rate, Borg dyspnoea scale score, and airway dryness score (mouth, nose, and throat) between the groups (Fig. 3 and Additional file 1: Table S1). During the study, no severe adverse events attributable to the randomization group were observed.

## Discussion

In this multicenter randomized clinical trial, we found that compared with COT, HFNC did not reduce need for intubation during hospitalization in non-acidotic patients with acute COPD exacerbation with mild hypercapnia; furthermore, it increased the length of hospital stay and hospital costs.

In this study, we found there was an insignificant difference in the proportion of patients who met the predetermined criteria for intubation between the HFNC and COT groups; however, it was significantly lower than previously expected value [6] in the COT group. This may be related to the significant improvement of clinical management of patients with acute COPD exacerbation in recent years, including the extensive use of NPPV during acute exacerbation, which have remarkably reduced the need for intubation [1–3]. Because of the low rate of intubation, consequently a difference on the primary outcome of need for intubation between the two groups was difficult to demonstrate.

However, it is noteworthy that the length of hospital stay and hospital cost in the HFNC group were significantly higher than those in the COT group. The length of hospital stay of the COT group in our study was similar to that reported in recent studies [29, 30] with comparable underling lung function. We found that the median duration from randomization to the start of NPPV treatment was longer in the HFNC group than in the COT group (4.0 vs. 2.0 days), which may delay the upgrade of NPPV treatment and increase the length of hospital stay. In addition, the lack of experience with this new technology among COPD patients on general respiratory wards may impact the length of hospital stay and hospital costs. Furthermore, it is worth noting that higher HFNC gas flow would lead to a large amount of oxygen consumption and an increase in medical cost. This factor should be considered especially in clinical scenarios with serious oxygen shortages because oxygen is not an inexhaustible resource. Therefore, these outcomes need to be investigated further in the future clinical studies.

Previous studies showed that HFNC was well tolerated in patients with acute hypoxic respiratory failure; however, its adherence in hypercapnia respiratory failure is rarely explored [20]. Similar to the report of Fraser, et al. [18], we also found that the tolerance of HFNC was not superior to COT, and intolerance (52%) was the main reason for the treatment failure in HFNC group. It may be related to the milder illness severity in our patients, which is similar to the intolerance of NPPV reported in patients with COPD exacerbation with mild hypercapnia [5, 7]. Therefore, the HFNC flow rate (median 30 L/min) adjusted according to patient’s maximum tolerance in our study was obviously lower than that used in patients with acute hypoxic respiratory failure (50 ~ 60 L/min) [14–16]. However, the HFNC flow rate was comparable to that in previous clinical physiological studies [20–22, 31–33] showing the physiological advantage of HFNC treatment in COPD patients.

Recently, Cortegiani et al. [34] found that HFNC and NPPV can equally reduce PaCO_2_ levels in patients with mild-to-moderate COPD exacerbation (pH 7.25–7.35; PaCO_2_ ≥ 55 mmHg) after 2 h of treatment. In our study, HFNC was not associated with lower PaCO_2_ levels and dyspnoea compared with COT. In addition to the lower HFNC flow rate than that of Cortegiani et al.’s study, another possible reason is that the baseline severity of hypercapnia (PaCO_2_) and the degree of dyspnoea (Borg score and respiratory rate) of the patients included in this study were significantly lower than those of patients in the above physiological study [17–20]. Recent studies have shown that individual responses to HFNC are heterogeneous in patients with acute COPD exacerbations [31], which may be related to the baseline PaCO_2_ level [35]. Therefore, whether the clinical outcomes of acute COPD exacerbation patients with acute respiratory acidosis (pH < 7.35) could benefit most from HFNC treatment needs to be explored further.

Another potential advantage of HFNC is that it may reduce the patient’s need for NPPV. The need for NPPV in our patients is similar to that in another study with a comparable underlying lung function (forced expiratory volume in one second % predicted about 32%) [29]. In this study, the NPPV rate was lower in the HFNC group than in the COT group; however, the difference was not statistically significant. In contrast, in a recently published randomized clinical trial, Li et al. [36] found that HFNC can significantly reduce the demand for NPPV compared to nasal prong in milder acute COPD exacerbation patients (forced expiratory volume in one second % predicted about 60%), and compared to our study, more patients needed to be supported with NPPV in Li et al.’s study (19.4% vs. 10.4%). The reasons for the differences between the two studies are difficult to explain clearly. However, compared with our study, patients in the Li et al. [36] were more severely ill during acute exacerbations with a higher APACHE II score (14.7 vs. 10.0) and lower oxygenation (PaO_2_ 55 vs. 70 mmHg). A recently published study has shown that HFNC is more effective than COT in patients with moderate acute hypoxic respiratory failure [14]. This may be one of the main reasons why the two studies have different results.

To our knowledge, this study is the largest muticenter clinical trial to explore the use of HFNC in patients with acute COPD exacerbation with mild hypercapnia. However, the present study has several limitations. First, the proportion of patients who met the criteria for intubation in our study was much lower than that the expected value [6], so the study power was limited. According to the results of our data, if the study reaches 80% efficacy and 0.05 significant, a larger sample size of 1474 is required to achieve a significant difference between the two treatment groups. Second, due to the nature of the technical characteristics of the intervention group, it was difficult to achieve a double-blind design. To mitigate this unavoidable bias, investigators were not involved in the clinical decision-making progress; besides, the database was controlled by a third party unaware of the study design and outcomes. Third, due to the difficulty of calculating the true cost of hospital admissions, the cost in the study was just a rough value. Finally, of 1276 patients with acute exacerbation of COPD with mild hypercapnia screened, 445 patients (35%) refused to participate in this study, and it might increase the selection bias. However, there was no significant difference in age, gender, SpO_2_, and arterial blood gas on admission between those who declined to participate and the enrolled patients (Additional file 3: Table S3).

## Conclusions

In this multicenter randomized controlled study, HFNC compared to COT did not reduce the need for intubation among patients with acute COPD exacerbation with mild hypercapnia; secondary analyses suggested HFNC increased the length of hospital stay and hospital costs. The future studies should focus on patients with acute COPD exacerbation with respiratory acidosis (pH < 7.35). However, because the primary outcome rate was well below expected, the study was underpowered to show a meaningful difference between the two treatment groups.

## Supplementary Information

**Additional file 1.** **Table S1:** Settings of treatment, vital sign, Borg scores, airway dryness score and blood gas analysis between the conventional oxygen therapy group and the high-flow nasal cannula group within 72 hours after randomization.

**Additional file 2.** **Table S2:** Comparison of daily duration of treatment with 7days after randomization, the time of treatment failure and noninvasive positive pressure ventilation start between the conventional oxygen therapy group and the high-flow nasal cannula group.

**Additional file 3.** **Table S3:** The comparison of age, gender, oxygenation and blood gas parameters on admission between the enrolled patients and the declined patients.

**Additional file 4.** **Figure S1:** Kaplan-Meier analysis of time since intervention to readmission during 90 days follow-up period. The cumulative incidence function and Gray’s test were used to consider deaths as competing events to evaluate the difference of time to readmission for acute exacerbation between two groups.

## Data Availability

The datasets used and/or analyzed during the current study are available from the corresponding author on reasonable request.

## Abbreviations

APACHE: Acute physiology and chronic health evaluation
COPD: Chronic obstructive pulmonary disease
COT: Conventional oxygen therapy
FiO_2_: Fraction of inspiration oxygen
HFNC: High-flow nasal cannula
IQR: Interquartile ranges
NPPV: Non-invasive positive pressure ventilation
PaCO_2_: Arterial partial pressure of carbon dioxide
PaO_2_: Arterial partial pressure of oxygen
SpO_2_: Oxygen saturation
