# Supplementary material for: High-flow nasal cannula versus conventional oxygen therapy in acute COPD exacerbation with mild hypercapnia: a multicenter randomized controlled trial
Source: Crit Care. 2022 Apr 15;26:109. doi: 10.1186/s13054-022-03973-7 (PMC9013098; doi:10.1186/s13054-022-03973-7)
Supplement: Supplementary file 1 — Additional file 1. Table S1: Settings of treatment, vital sign, Borg scores, airway dryness score and blood gas analysis between the conventional oxygen therapy group and the high-flow nasal cannula group within 72 hours after randomization. [file 13054_2022_3973_MOESM1_ESM.docx]

**Table E1:** Settings of treatment, vital sign, Borg scores, airway dryness score and blood gas analysis between conventional oxygen therapy group and high flow nasal oxygen group within 72hours after randomization.

| Characteristic | 2 hours after randomization | | *P^a^* | 24 hours after randomization | | *P^a^* | 48 hours after randomization | | P*^a^* | 72 hours after randomization | | *P^a^* |
| --- | --- | --- | --- | --- | --- | --- | --- | --- | --- | --- | --- | --- |
|  | HFNC  group | COT  group |  | HFNC  group | COT  group |  | HFNC  group | COT  group |  | HFNC  group | COT  group |  |
| Flow, median (IQR), L/min | 30.0  (25.0- 40.0) | 2.0  (2.0-3.0) |  | 30.0  (25.0- 40.0) | 2.0  (2.0-3.0) |  | 30.0  (25.0- 40.0) | 2.0  (2.0-3.0) |  | 35.0  (25.0- 40.0) | 2.0  (2.0-3.0) |  |
| FiO_2_, median (IQR) | 0.32  (0.29-0.40) | - | - | 0.33  (0.30-0.40) | - | - | 0.33  (0.30-0.40) | - | - | 0.33  (0.30-0.40) | - | - |
| Gas temperature, median (IQR) , ℃ | 34.0  (31.0-34.0) | - | - | 34.0  (31.0-34.0) | - | - | 34.0  (31.0-34.0) | - | - | 34.0  (31.0-34.0) | - | - |
| Respiratory rate, median (IQR), breaths/min | 20.0  (19.0-22.0) | 20.0  (20.0-22.0) | 0.402 | 20.0  (19.0-21.0) | 20.0  (19.0-21.0) | 0.920 | 20.0  (19.0-21.0) | 20.0  (19.0-21.0) | 0.566 | 20.0  (19.0-21.0) | 20.0  (18.0-21.0) | 0.485 |
| Heart rate, median (IQR), beats/min | 85.0  (78.0-96.0) | 85.0  (78.0-94.0) | 0.898 | 82.0  (78.0-90.0) | 82.0  (78.0-90.0) | 0.848 | 80.0  (78.0-90.0) | 81.0  (76.0-88.0) | 0.591 | 82.0  (76.0-89.0) | 80.0  (76.0-88.0) | 0.447 |
| Mean blood pressure, median (IQR), mmHg | 93.3  (86.7-98.0) | 94.7  (89.0-100.0) | 0.212 | 91.3  (86.0-97.0) | 91.0  (85.3-97.3) | 0.732 | 90.3  (83.3-96.7) | 92.7  (86.0-97.7) | 0.210 | 91.0  (83.0-97.3) | 91.3  (86.7-97.0) | 0.651 |
| SpO_2_, median (IQR), (%) | 95.0  (92.0-97.0) | 95.0  (92.0-97.0) | 0.533 | 95.0  (93.0-97.0) | 95.0  (92.0-97.0) | 0.355 | 96.0  (94.0-98.0) | 95.0  (93.0-98.0) | 0.602 | 96.0  (93.0-98.0) | 95.7  (93.5-98.0) | 0.662 |
| Borg scale score | 4.0  (2.0-5.0) | 4.0  (3.0-5.0) | 0.882 | 3.0  (2.0-5.0) | 3.0  (2.0-4.0) | 0.853 | 3.0  (2.0-4.0) | 3.0  (2.0-4.0) | 0.387 | 2.0  (2.0-3.5) | 2.0  (2.0-4.0) | 0.735 |
| Mouth dryness score  median (IQR) | 3.0  (1.0-4.0) | 3.0  (1.0-4.0) | 0.436 | 3.0  (1.0-4.0) | 2.0  (1.0-4.0) | 0.717 | 2.0  (1.0-4.0) | 2.0  (1.0-4.0) | 0.660 | 2.0  (0.0-3.0) | 2.0  (1.0-4.0) | 0.347 |
| Nasal dryness score  median (IQR) | 3.0  (1.0-4.0) | 3.0  (1.0-4.0) | 0.500 | 2.0  (1.0-4.0) | 2.0  (1.0-4.0) | 0.734 | 2.0  (1.0-4.0) | 2.0  (1.0-4.0) | 0.143 | 2.0  (1.0-3.0) | 2.0  (1.0-4.0) | 0.110 |
| Throat dryness score  median (IQR) | 3.0  (1.0-4.0) | 2.0  (1.0-4.0) | 0.664 | 2.0  (1.0-4.0) | 2.0  (1.0-4.0) | 0.888 | 2.0  (1.0-3.5) | 2.0  (1.0-4.0) | 0.639 | 2.0  (1.0-3.0) | 2.0  (1.0-4.0) | 0.234 |
| pH  median (IQR) | 7.39  (7.36-7.43) | 7.40  (7.37-7.42) | 0.559 | 7.40  (7.37-7.43) | 7.39  (7.35-7.42) | 0.019 | 7.40  (7.36-7.43) | 7.40  (7.37-7.42) | 0.689 | 7.40  (7.38-7.43) | 7.40  (7.37-7.43) | 0.429 |
| PaCO_2_, median (IQR), mmHg | 50.8  (46.6-58.0) | 51.3  (46.6-57.0) | 0.610 | 50.8  (45.2-57.5) | 51.9  (46.5-59.3) | 0.197 | 50.3  (45.0-58.0) | 50.0  (45.1-57.0) | 0.726 | 49.1  (45.0-56.7) | 49.5  (45.0-55.2) | 0.926 |
| PaO_2_, median (IQR), mmHg | 75.0  (62.0-87.0) | 76.0  (62.0-91.0) | 0.856 | 75.0  (64.0-89.2) | 73.9  (62.0-86.4) | 0.306 | 78.0  (63.0-93.4) | 75.0  (66.3-87.9) | 0.493 | 82.0  (65.2-102.5) | 80.0  (68.0-92.0) | 0.484 |
| PaO_2_/FiO_2_, median (IQR), mmHg | 223.2  (173.8-266.7) | - | - | 231.3  (182.9-300.0) | - | - | 228.0  (187.5-302.5) | - | - | 240.0  (194.1-297.5) | - | - |
| Lactic acid, median (IQR), mmol/L | 1.2  (1.0-1.7) | 1.1  (0.8-1.6) | 0.511 | 1.3  (0.9-1.8) | 1.3  (1.0-2.2) | 0.166 | 1.4  (1.0-1.9) | 1.5  (1.0-2.0) | 0.360 | 1.3  (1.0-1.8) | 1.5  (0.8-1.9) | 0.413 |

Abbreviations: COT=conventional oxygen therapy, FiO_2_= fraction of inspired oxygen, HFNC= high-flow nasal cannula, IQR= interquartile range, PaCO_2_= arterial partial pressure of carbon dioxide, PaO_2_=arterial partial pressure of oxygen, SpO_2_= oxygen saturation.

^a^ Mann-Whitney U test
