# Supplementary material for: High-flow nasal cannula versus conventional oxygen therapy in acute COPD exacerbation with mild hypercapnia: a multicenter randomized controlled trial
Source: Crit Care. 2022 Apr 15;26:109. doi: 10.1186/s13054-022-03973-7 (PMC9013098; doi:10.1186/s13054-022-03973-7)
Supplement: Supplementary file 2 — Additional file 2. Table S2: Comparison of daily duration of treatment with 7days after randomization, the time of treatment failure and noninvasive positive pressure ventilation start between the conventional oxygen therapy group and the high-flow nasal cannula group. [file 13054_2022_3973_MOESM2_ESM.docx]

**Table E2:** Comparison of daily duration of treatment with 7days after randomization, the time of treatment failure and noninvasive positive pressure ventilation start between high flow nasal cannula group and conventional oxygen therapy group.

| Characteristic | high flow nasal cannula group  (n=158) | Conventional oxygen therapy group  (n=172) | *P^a^* |
| --- | --- | --- | --- |
| Duration of treatment at day 1*, NO.  Median (IQR), hours | 158  10.0 (6.0-18.0) | 172  15.0 (8.5-22.0) | <0.001 |
| Duration of treatment at day 2, NO.  Median (IQR), hours | 156  18.0 (10.0-24.0) | 165  20.0(12.0-24.0) | 0.090 |
| Duration of treatment at day 3, NO.  Median (IQR), hours | 150  18.0(10.0-24.0) | 157  20.0(12.0-24.0) | 0.045 |
| Duration of treatment at day 4, NO.  Median (IQR), hours | 139  17.0(10.0-24.0) | 153  20.0(12.0-24.0) | 0.058 |
| Duration of treatment at day 5, NO.  Median (IQR), hours | 115  18.0(12.0, 24.0) | 144  18.0(10.0-24.0) | 0.342 |
| Duration of treatment at day 6, NO.  Median (IQR), hours | 107  16.0(10.0-24.0) | 145  19.0(12.0-24.0) | 0.162 |
| Duration of treatment at day 7, NO.  Median (IQR), hours | 94  15.0(10.0-24.0) | 126  17.50(10.0-24.0) | 0.748 |
| Total duration of treatment within 3 days after randomization, median (IQR), hours | 45.0  (28.0-60.0) | 52.0  (32.0-63.0) | 0.027 |
| Total duration of treatment within 7 days after randomization, median (IQR), hours | 82.0  (44.0-137.0) | 111.0  (66.0-148.5) | 0.005 |
| Duration from randomization to the failure treatment, median (IQR), hours | 3.0  (2.0-5.0) | 2.0  (2.0-5.0) | 0.560 |
| Duration from randomization to the start of NPPV treatment, median (IQR), hours | 4.0  (3.0-8.0) | 2.0  (1.0-5.0) | 0.060 |
| Duration from admission to start on randomized therapy, median (IQR), days | 0  (0-1) | 0  (0-1) | 0.972 |

Abbreviations: IQR= interquartile range, NPPV= noninvasive positive pressure ventilation.

* Day 1 refers to the day of randomization.

^a^ Mann-Whitney U test
