# Supplementary material for: High-flow nasal cannula versus conventional oxygen therapy in acute COPD exacerbation with mild hypercapnia: a multicenter randomized controlled trial
Source: Crit Care. 2022 Apr 15;26:109. doi: 10.1186/s13054-022-03973-7 (PMC9013098; doi:10.1186/s13054-022-03973-7)
Supplement: Supplementary file 3 — Additional file 3. Table S3: The comparison of age, gender, oxygenation and blood gas parameters on admission between the enrolled patients and the declined patients. [file 13054_2022_3973_MOESM3_ESM.docx]

**Table E3** The comparison of age, gender, oxygenation and blood gas parameters on admission between enrolled patients and declined patients.

| Characteristics of the patients at admission | Enrolled patients  (n=330) | Declined patients  (n=410) | *P* | |
| --- | --- | --- | --- | --- |
| Age, median (IQR), y | 69.5(64.0-75.0) | 70.0(64.0-76.0) | 0.449 |  |
| Men, No. (%) | 277(83.9%) | 344(83.9%) | 0.989 |  |
| SpO_2_, median (IQR), % | 93.0(88.6-96.0) | 93.1(90.0-96.5) | 0.361 |  |
| pH, median (IQR), units | 7.40(7.37-7.42) | 7.40(7.37-7.43) | 0.373 |  |
| PaCO_2_, median (IQR), mmHg | 51.0(47.5-57.5) | 51.3(47.5-59.6) | 0.331 |  |
| PaO_2_, median (IQR), mmHg | 69.0(57.0-83.3) | 69.5(55.0-88.0) | 0.823 |  |
| Bicarbonate, median (IQR), mmol/L | 31.3(28.3-34.9) | 31.8(29.0-35.2) | 0.890 | |
