# Supplementary material for: High-flow nasal cannula versus conventional oxygen therapy in acute COPD exacerbation with mild hypercapnia: a multicenter randomized controlled trial
Source: Crit Care. 2022 Apr 15;26:109. doi: 10.1186/s13054-022-03973-7 (PMC9013098; doi:10.1186/s13054-022-03973-7)
Supplement: Supplementary file 4 — Additional file 4. Figure S1: Kaplan-Meier analysis of time since intervention to readmission during 90 days follow-up period. The cumulative incidence function and Gray’s test were used to consider deaths as competing events to evaluate the difference of time to readmission for acute exacerbation between two groups. [file 13054_2022_3973_MOESM4_ESM.docx]

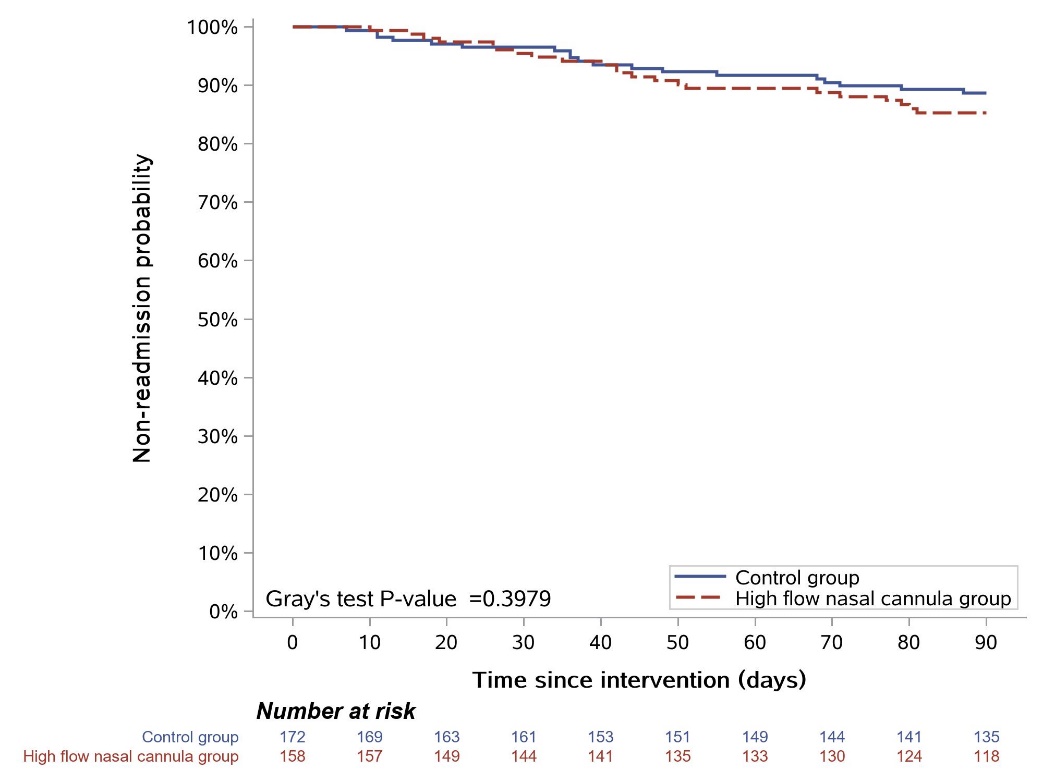


**Figure E1** Kaplan-Meier analysis of time since intervention to readmission during 90 days follow-up period. The cumulative incidence function and Gray’s test were used to consider deaths as competing events to evaluate the difference of time to readmission for acute exacerbation between two groups.
